# Bernard Witholt, 1941–2015: A Great Microbial Biotechnologist

**DOI:** 10.1111/1751-7915.12293

**Published:** 2015-06-15

**Figure:**
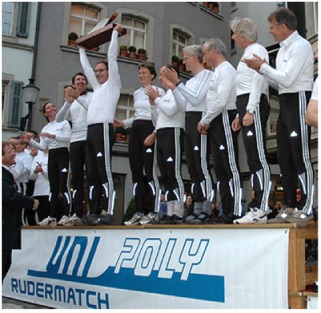
Photo courtesy of ETH Life and Akademischer Sportverband Zürich (ASVZ).

## Personal tributes from some researchers he influenced

